# Puerarin Protects Myocardium From Ischaemia/Reperfusion Injury by Inhibiting Ferroptosis Through Downregulation of VDAC1

**DOI:** 10.1111/jcmm.70313

**Published:** 2024-12-27

**Authors:** Fajia Hu, Tie Hu, Andi He, Yong Yuan, Xiuqi Wang, Chenchao Zou, Yamei Qiao, Huaihan Xu, Lanxiang Liu, Qun Wang, Jichun Liu, Songqing Lai, Huang Huang

**Affiliations:** ^1^ Institute of Cardiovascular Surgical Diseases, the First Affiliated Hospital, Jiangxi Medical College Nanchang University Nanchang Jiangxi China; ^2^ Department of Cardiovascular Surgery, the Second Affiliated Hospital, Jiangxi Medical College Nanchang University Nanchang Jiangxi China; ^3^ School of Pharmacy, Jiangxi Medical College Nanchang University Nanchang Jiangxi China; ^4^ Department of Cardiovascular Surgery, the First Affiliated Hospital, Jiangxi Medical College Nanchang University Nanchang Jiangxi China

**Keywords:** ferroptosis, mitochondria, myocardial ischaemia–reperfusion injury, puerarin, VDAC1

## Abstract

Despite improvements in interventional techniques leading to faster myocardial reperfusion postmyocardial infarction, there has been a significant rise in the occurrence of myocardial ischaemia/reperfusion injury (MI/RI). A deeper understanding of the underlying mechanisms of MI/RI could offer a crucial approach to reducing myocardial damage and enhancing patient outcomes. This study examined the myocardial protective properties of puerarin (PUE) in the context of MI/RI using hypoxia/reoxygenation (H/R) or ischaemia/reperfusion (I/R) injury models were employed in H9c2 cells and C57BL/6 mice. Our findings demonstrate that pretreatment with PUE effectively mitigated cardiomyocyte ferroptosis, restored redox balance, preserved mitochondrial energy production and maintained mitochondrial function following MI/RI. Furthermore, these cardioprotective effects of PUE were found to be mediated by the downregulation of voltage‐dependent anion channel 1 (VDAC1) protein. These data reveal a novel mechanism by which PUE inhibits MI/RI and reveal that this protective effect of PUE is dependent on the downregulation of VDAC1.

**Abbreviations**
Fer‐1: ferrostatin‐1
LAD: left anterior descending branch
LVEF: left ventricular ejection fraction
LVFS: left ventricular fractional shortening
MMP: mitochondrial membrane potential
mPTP: mitochondrial permeability transition
PUE: puerarin
TCA: tricarboxylic acid
TTC: 2,3,5‐triphenyltetrazolium chloride
VDAC1: voltage‐dependent anion channel 1

## 1 Introduction

Acute myocardial infarction (AMI) is a serious cardiovascular disease that people all over the world need to face together [1]. The most effective treatment now is to restore myocardial perfusion as early as possible. Unfortunately, with the restoration of perfusion occurs myocardial ischaemia–reperfusion injury (MI/RI), a serious complication that further damages the myocardium [2]. Previous studies have suggested that the mechanism of MI/RI is related to apoptosis, pyroptosis, necroptosis, autophagy, disturbed energy metabolism, intracellular calcium overload, cellular inflammation and oxidative stress [3, 4, 5]. Although several recent studies of MI/RI have targeted ROS and oxidative stress [6, 7], the exact mechanisms are not well understood. In‐depth studies of these mechanisms could help to identify new potential therapeutic targets.

Ferroptosis is a recently discovered mechanism of programmed cell death triggered by dysregulated iron‐dependent lipid peroxidation [8]. Numerous studies have indicated that MI/RI results in oxidative stress, subsequently compromising mitochondrial function and serving as a primary mechanism for MI/RI [9]. It is noteworthy that ferroptosis is distinguished by the production of reactive oxygen species (ROS) resulting from the build‐up of iron and lipid peroxidation, ultimately leading to cellular demise. An increasing number of studies have revealed that ferroptosis is involved in various physiological and pathological processes of MI/RI [10, 11].

Puerarin (PUE) is the main active ingredient in the leguminous traditional Chinese medicine *Pueraria mirifica* [12]. Modern pharmacological studies have shown that PUE has a variety of biological activities such as anti‐inflammatory, antioxidant response, antiapoptosis, oestrogen‐like activity, control of blood pressure and autophagy regulation [13, 14, 15, 16, 17]. A study suggests that PUE exerts a protective effect against MI/RI by inhibiting NLRP3 inflammatory vesicles [18]. Recent research has indicated that flavonoids, particularly PUE, exhibit protective effects against MI/RI through various mechanisms [19]. Multiple research studies have indicated that PUE may play a role in safeguarding against a range of diseases through the preservation of mitochondrial function [20, 21, 22]. Voltage‐dependent anion channel 1 (VDAC1), serving as a crucial modulator of mitochondrial activity, is widely distributed throughout the outer mitochondrial membrane [23]. Prior research has demonstrated that certain herbal monomers possess the ability to facilitate VDAC1‐mediated protection of cardiomyocytes from damage caused by MI/RI [24].

This study provides novel insights into the mechanism by which PUE preconditioning confers protection against MI/RI. Our findings demonstrate that PUE preconditioning reduces the expression of VDAC1 and attenuates ferroptosis in the context of MI/RI.

## 2 Materials and Methods

### 2.1 Cells and Animals

The H9c2 cell line was acquired from the Cell Bank/Stem Cell Bank in Beijing, China, and cultured in high‐glucose Dulbecco's modified Eagle medium (H‐DMEM; Hyclone, GE Healthcare Life Science, Pittsburgh, PA, USA) supplemented with 10% foetal bovine serum (Gibco, Thermo Fisher Scientific, Waltham, MA, USA), 100 U/mL penicillin and 100 μg/mL streptomycin (Servicebio Technology Co. Ltd., Wuhan, China). The cells were maintained in a humidified incubator at 37°C with a humidity level of 95%, oxygen concentration of 21% and CO_2_ concentration of 5%.

Male C57BL/6 mice in good health, between 6 and 8 weeks old, were acquired from the Animal Center at Nanchang University in China. The experimental protocol followed the guidelines set forth by the National Institutes of Health (NIH) and was approved by the Animal Experimentation Ethics Committee of The First Affiliated Hospital of Nanchang University (No. CDYFY‐IACUC‐202209QR004). The mice were housed in a controlled environment with a temperature of 23°C ± 2°C, humidity of 50% ± 5% and a 12‐h light–dark cycle.

### 2.2 Reagents and Antibodies

PUE (purity≥ 99%) and ferrostatin‐1 (Fer‐1, ferroptosis inhibitor) were purchased from MedChemExpress (Shanghai, China). Adenovirus AD/NC and AD/VDAC1 were obtained from Gene Pharma Co. Ltd. (Suzhou, China). Primary antibodies against GPX4 and β‐actin were bought from ZenBio Science (Chengdu, Sichuan, China). Primary antibodies against VDAC1 and PTGS2 were obtained from Proteintech (Chicago, IL, USA). ZenBio Science (Chengdu, Sichuan, China) supplied anti‐rabbit IgG and anti‐mouse IgG.

### 2.3 In Vivo Experiments

#### 2.3.1 Construction of the I/R Model

As outlined in our prior research, the in vivo I/R model was established through transient ligation of the left coronary artery [25]. After inducing anaesthesia with 3% isoflurane, mice were placed in a supine position and maintained under 1.5% isoflurane. A thoracotomy was then conducted at the fourth intercostal space to expose the heart by opening the pericardium. Closure of the left anterior descending artery (LAD) was accomplished using a 4–0 silk suture and a snare was created by threading a short polyethylene tube through the suture ends. The snare was applied to the heart surface to induce ischaemia and subsequently removed to allow for reperfusion. Mice in the sham group underwent a similar procedure without occlusion of the LAD. Mice hearts underwent 60 min of ischaemia, then 24 h of reperfusion to mimic in vivo myocardial infarction/reperfusion injury.

#### 2.3.2 In Vivo Experimental Grouping

The mice were then randomly allocated into four distinct groups: (1) sham group; (2) I/R group; (3) I/R + PUE group; and (4) I/R + Fer‐1 group. Mice in the sham and I/R groups received normal saline, while mice in the I/R + PUE group were administered 100 mg/kg PUE intragastrically for a duration of 21 days [26]. Additionally, mice in the I/R + Fer‐1 group were intraperitoneally injected with 1 mg/kg Fer‐1 for a period of 2 weeks.

#### 2.3.3 Assessment of Infarct Size

The extent of myocardial infarction was assessed using triphenyl tetrazolium chloride (TTC) staining. After a 24‐h period of reperfusion, the LAD was blocked. Afterwards, the hearts were sliced into 8 μm sections and treated with 2% TTC from Solarbio Life Sciences in China, then incubated at 37°C for 20 min following a quick freeze in liquid nitrogen. Digital images were captured after fixing the tissue sections in 4% paraformaldehyde for 24 h.

#### 2.3.4 Determination of LDH and CK‐MB

Following various treatments, serum samples were collected from each group after blood collection and centrifugation for 10 min at 4°C. Subsequently, the serum levels of LDH and CK‐MB were assessed using specific detection kits (Nanjing Jiancheng Bioengineering Institute, Nanjing, China) according to the manufacturer's guidelines.

#### 2.3.5 Assessment of Cardiac Function

Following reperfusion, cardiac function was assessed utilising two‐dimensional transthoracic echocardiography with the Vevo2100 imaging system (VisualSonics Inc., Canada) on anaesthetised mice using 1.5% isoflurane. An operator who was unaware of the study performed the measurements.

#### 2.3.6 Dihydroethidium (DHE) Staining

DHE staining was performed in accordance with the guidelines provided by the manufacturer. Specifically, frozen heart tissues were sectioned into 8‐μm cross‐axis slices and subsequently exposed to DHE solution (Servicebio, Wuhan, China) at 37°C for a duration of 1 h. Subsequently, fluorescent images were captured utilising a Nikon fluorescent microscope (Nikon, Tokyo, Japan).

#### 2.3.7 TUNEL Staining

After receiving different treatments, samples from the heart were gathered and preserved in 4% paraformaldehyde, then dehydrated in sucrose and finally, enclosed in optimal cutting temperature (OCT) compound. Afterwards, the hearts were cut into 8 μm slices, treated with paraformaldehyde, permeabilised using 1% Triton X‐100 and then exposed to a working buffer (One‐Step TUNEL Apoptosis Assay Kit, Beyotime, China) for 1 h at 37°C following the instructions provided by the manufacturer. After sealing the slides with glycerol, they were examined using a Nikon fluorescent microscope from Tokyo, Japan.

#### 2.3.8 Evaluation of MDA and Total Iron Levels

MDA and total iron levels in left ventricular tissue homogenates were measured with specialised detection kits from Nanjing Jiancheng Bioengineering Institute in Nanjing, China, according to the manufacturer's instructions.

### 2.4 In Vitro Experiments

#### 2.4.1 Adenovirus Transfection and H/R Injury

H9c2 cells were transfected with either AD‐VDAC1 or AD‐NC and cultured in H‐DMEM with 10% FBS. Following a 48‐h incubation period, the transfection efficiency was determined to be approximately 85%, after which additional experiments were conducted.

As mentioned in the previous study [27], cells were cultured in anoxia fluid (CaCl_2_ 1.0 mM, HEPES 20 mM, KCl 10 mM, MgSO_4_ 1.2 mM, NaCl 98.5 mM, NaH_2_PO_4_ 0.9 mM, NaHCO_3_ 36 mM, sodium lactate 40 mM and pH 6.8) and reoxygenation fluid (CaCl_2_ 1.0 mM, glucose 5.5 mM, HEPES 20 mM, KCl 5 mM, MgSO_4_ 1.2 mM, NaCl 129.5 mM, NaH_2_PO_4_ 0.9 mM, NaHCO_3_ 20 mM and pH 7.4). The H9c2 cells in Petri dishes were then placed in an air‐tight anoxic chamber at 37°C with a gas mixture of 95% N_2_ and 5% CO_2_ for 3 h, followed by a change to 95% O_2_ and 5% CO_2_ for 2 h to induce H/R injury.

### 2.5 In Vitro Experimental Grouping

H9c2 cardiomyocytes were categorised into distinct experimental groups as follows: (1) Control group: H9c2 cells were cultured under standard conditions; (2) H/R group: H9c2 cells were subjected to H/R injury; (3) concentration–effect PUE group: H9c2 cells pretreated with varying concentrations of PUE (2.5, 5, 10, 20, 40 and 80 μM) for 48 h; (4) concentration–effect PUE + H/R group: H9c2 cells were pretreated with PUE (2.5, 5, 10, 20, 40 and 80 μM) for 48 h prior to H/R injury; (5) PUE + H/R group: H9c2 cells were pretreated with 10 μM PUE for 48 h before H/R injury; (6) Fer‐1 + H/R group: H9c2 cells were pretreated with 5 μM ferrostatin‐1 before H/R injury; (7) AD‐VDAC1 + PUE + H/R group: H9c2 cells were transfected with AD‐VDAC1 for 48 h and pretreated with 10 μM PUE for 48 h before H/R injury; and (8) AD‐NC + PUE + H/R group: H9c2 cells were transfected with AD‐NC for 48 h and pretreated with PUE for 48 h before H/R injury.

### 2.6 Cell Viability and LDH Activity Assay

Cell viability was assessed using the Cell Counting Kit‐8 (CCK‐8, Good Laboratory Practice Bioscience, Montclair, CA, USA) and LDH (Beyotime, Shanghai, China) levels in accordance with the manufacturer's guidelines.

### 2.7 Detection of Oxidative Stress

Intracellular levels of ROS were measured using a ROS detection kit from Beyotime in H9c2 cells. Cells were exposed to DCFH‐DA for 20 min at 37°C without light exposure. Subsequently, the levels of ROS within each experimental group were visualised utilising an inverted fluorescence microscope (Olympus, Tokyo, Japan).

Following various treatments, H9c2 cells were subjected to digestion with 0.25% trypsin and sonication to obtain cell homogenates. Afterwards, the mixtures were spun at 12,000 × *g* at 4°C for 15 min, leading to the retrieval of the liquid above the sediment. The liquid above the sediment was used to measure the amounts of GSH, GSSG, MDA and 4‐HNE following the instructions provided in the GSH and GSSG test kit, MDA test kit (Beyotime, Shanghai, China) and 4‐HNE test kit (Jianglai biology, Shanghai).

### 2.8 Flow Cytometry Assay

Mitochondrial permeability transition (mPTP), mitochondrial membrane potential (MMP) and apoptosis were assessed using the mPTP assay kit, the JC‐1 MMP assay kit and the Annexin V‐FITC apoptosis assay kit (BestBio, Shanghai, China), respectively. The detection of mPTP involved incubating the cell suspension with BbcellProbe M61 and quencher for 15 min at 37°C in the dark, followed by centrifugation and washing. The mPTP level was then immediately determined using a Cytomics FC 500 flow cytometer (excitation (Ex) = 488 nm and emission (Em) = 558 nm) (Beckman Coulter, Brea, CA, USA). The MMP of H9c2 cardiomyocytes was assessed by incubating the cells with JC‐1 dye at 37°C for 30 min in the absence of light, followed by centrifugation, washing and analysis using a Cytomics FC 500 flow cytometer with emission filters set at 530/580 nm (red) and 485/530 nm (green). To evaluate apoptosis, the cells were treated with 5 μL of V‐FITC membrane‐bound protein and 10 μL of propidium iodide (PI) for 20 min at 4°C and then analysed using a Cytomics FC 500 flow cytometer with excitation at 488 nm and emission at 578 nm.

### 2.9 Evaluation of Intracellular Lipid ROS and Ferrous Ions

Intracellular lipid ROS and ferrous iron levels were assessed using C11‐BODIPY581/591 and FerroOrange fluorescence, following the provided guidelines. H9c2 cells were subjected to the described treatment and then exposed to particular stains for 30 min at 37°C without light exposure. Afterwards, the levels of lipid ROS and ferrous iron inside the cells were measured with a fluorescence microscope.

### 2.10 Total Iron and Caspase‐3 Activity Assay

After being treated, H9c2 heart muscle cells from different test groups were digested using 0.25% trypsin, then washed twice with PBS and finally, lysed with sonication to ensure cellular homogenisation. The resulting homogenates were centrifuged to obtain supernatants, and the determination of total iron levels was conducted in accordance with the provided kit guidelines (Nanjing Jiancheng).

Caspase‐3 activity was evaluated following the guidelines provided by the manufacturer for the caspase‐3 assay kit from Beyotime in Shanghai, China. A mixture containing reaction buffer, homogenised cell samples from each experimental group and caspase‐3 substrate was prepared and dispensed into 96‐well plates, followed by a 2‐h incubation at 37°C. Afterwards, the amount of light absorbed was measured at a wavelength of 405 nm with a microplate reader. Concurrently, the protein concentrations in each group were determined utilising the Bradford method. In the end, the calculation of caspase‐3 activity was determined using these measurements.

### 2.11 Western Blot Analysis

The entirety of proteins derived from H9c2 cells and myocardial lysates underwent hydrolysis in RIPA lysis buffer (Beyotime, Shanghai, China). The quantification of protein concentration was achieved through utilisation of the BCA protein assay kit (Good Laboratory Practice Bioscience, Montclair, CA, USA). Subsequently, 20 μg of proteins were segregated via 10% or 12% sodium dodecyl sulphate–polyacrylamide gel electrophoresis (SDS‐PAGE) and subsequently transferred onto polyvinylidene fluoride (PVDF) membranes. The membranes were blocked and subjected to incubation with antibodies (diluted at a ratio of 1:1000) targeting VDAC1, GPX4, PTGS2 and β‐actin at a temperature of 4°C for an overnight duration. Subsequently, the membranes underwent incubation with horseradish peroxidase‐conjugated secondary antibodies. β‐Actin was utilised as the internal control. Finally, the protein bands were assessed utilising the Image J v1.5.3 software.

### 2.12 Statistical Analysis

The statistical software GraphPad Prism (La Jolla, CA, USA) was employed to perform an analysis of variance, presenting results in the form of mean ± standard deviation (SD). Statistical significance was defined as a *p*‐value less than 0.05.

## 3 Results

### 3.1 PUE Preconditioning Protects Cardiomyocytes From I/R Injury

**FIGURE 1:**
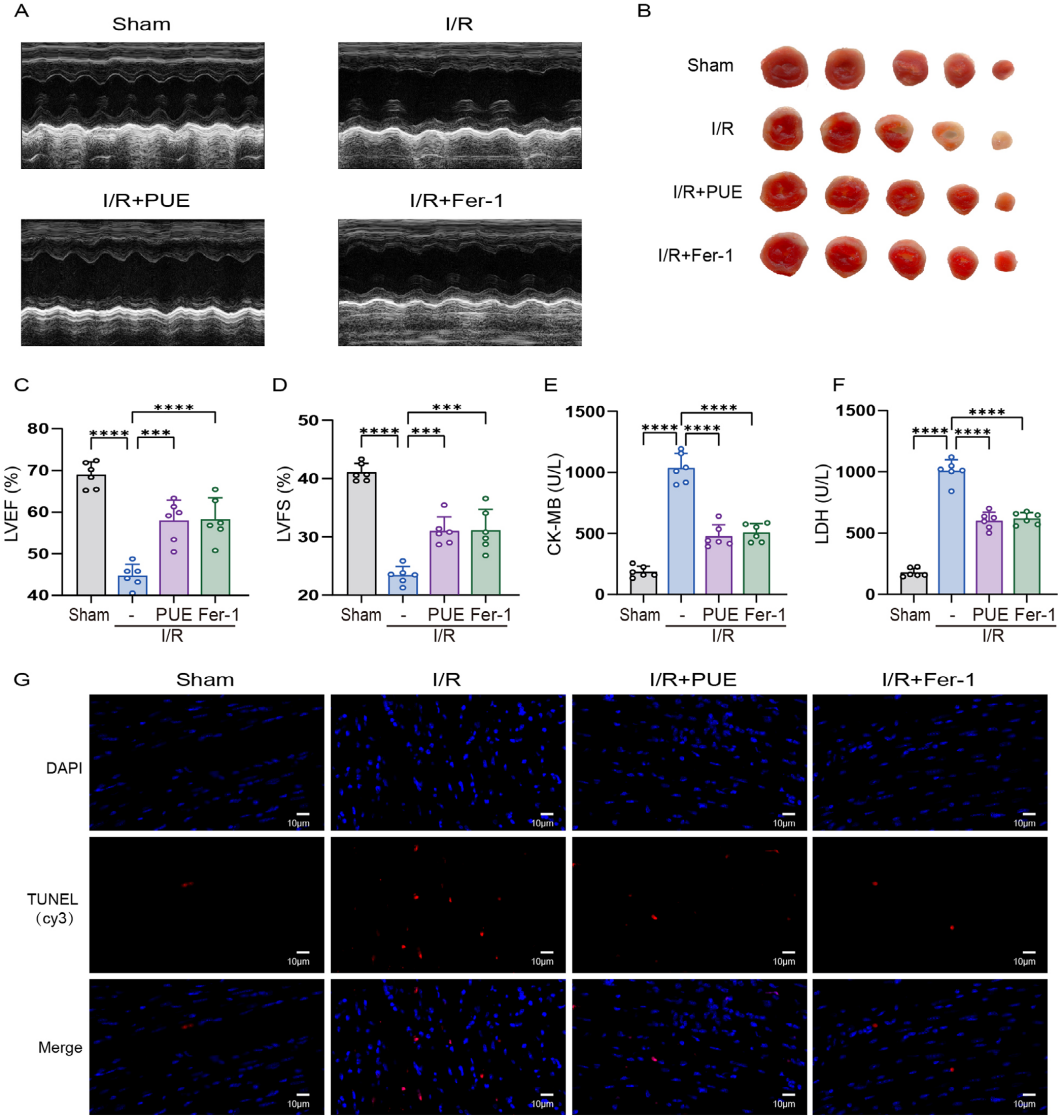
PUE preconditioning protects cardiomyocytes from I/R injury. (A) Representative images show the changes in cardiac structure detected by echocardiography in mice. (B) Representative images of infarct size were assessed using TTC staining. (C) Histogram of LVEF. (D) Histogram of LVFS. (E) Histogram of CK‐MB. (F) Histogram of LDH activity. (G) Representative images of TUNEL staining for assessment of cardiac injury (magnification, ×700). Data are expressed as the mean ± SD (*n* = 3 or 6). ***p < 0.001, ****p < 0.0001.

To evaluate the impact of PUE on MI/RI, we developed an I/R model in mice by occluding the anterior descending branch of the left coronary artery for a duration of 60 min. Following this, the relevant indices were assessed after a 24‐h reperfusion period [10, 27]. Our study revealed a significant increase in myocardial infarct area following I/R injury, as well as elevated levels of serum CK‐MB and LDH (Figure 1B,E,F). Additionally, echocardiographic findings demonstrated a significant decrease in left ventricular ejection fraction (LVEF) and left ventricular fractional shortening (LVFS) (Figure 1A,C,D). Following I/R injury, TUNEL staining revealed a notable increase in positive cells (Figure 1G). Pretreatment with PUE significantly mitigated the aforementioned enzymatic, morphological and functional alterations in mice following I/R injury. To investigate the potential role of ferroptosis in I/R injury, a cohort of mice was pretreated with Fer‐1, a known inhibitor of ferroptosis [28]. Our findings indicate that Fer‐1 and PUE exhibited comparable protective properties by mitigating the enzymatic, morphological and functional alterations associated with I/R injury.

### 3.2 PUE Pretreatment Attenuates Myocardial Ferroptosis After I/R Injury in Mice by Downregulating VDAC1

**FIGURE 2:**
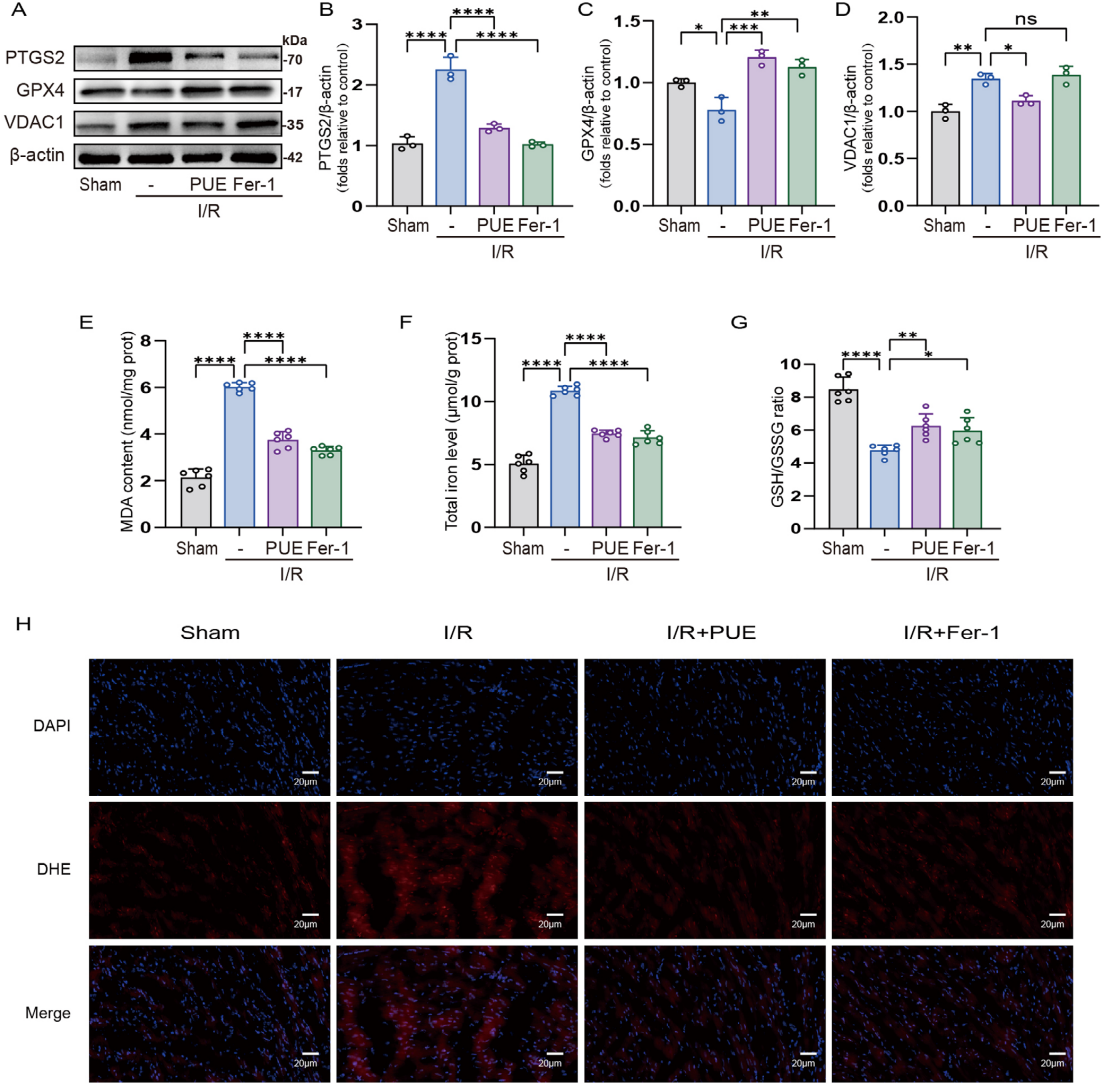
PUE pretreatment attenuates myocardial ferroptosis after I/R injury in mice by downregulating VDAC1. (A) Western blot detection of PTGS2 protein, GPX4 protein and VDAC1 protein expression in mouse myocardial lysates. (B, C, D) Histogram of PTGS2 protein, GPX4 protein and VDAC1 protein expression. (E) MDA level in mouse myocardial lysates. (F) Total iron level in mouse myocardial lysates. (G) GSH and GSSG ratios in mouse myocardial lysates. (H) The image of the myocardium was stained with DHE (magnification, ×400). Data are expressed as the mean ± SD (*n* = 3 or 6). ns, nonsignificant, *p < 0.05, **p < 0.01, ***p < 0.001, ****p < 0.0001.

To further explore the effect of PUE on ferroptosis in the context of I/R injury and its underlying mechanisms, we evaluated the protein expression levels of PTGS2 and GPX4, which are molecular markers of ferroptosis [29]. Our findings demonstrated a significant increase in PTGS2 protein levels following I/R injury, accompanied by a decrease in GPX4 levels. Importantly, treatment with either PUE or Fer‐1 reversed these alterations in protein expression (Figure 2A–C). Prior research has demonstrated a strong association between ferroptosis and lipid peroxidation as well as iron overload [30]. In this study, we conducted an analysis of lipid peroxidation and iron overload levels in each experimental group. Our findings revealed a significant increase in levels of MDA and total iron, along with a decrease in the GSH/GSSG ratio following I/R injury. Importantly, pretreatment with PUE or Fer‐1 successfully reversed the effects induced by I/R (Figure 2E–G). Furthermore, an examination of intracellular DHE levels in cardiomyocytes revealed that pretreatment with PUE or Fer‐1 resulted in significant inhibition of DHE levels induced by I/R injury (Figure 2H). Previous studies have shown that many types of herbal medicines can attenuate I/R injury by targeting VDAC1 [24, 31, 32]. To verify whether PUE targets VDAC1, we examined the protein expression of VDAC1 and observed a significant increase in VDAC1 levels post‐I/R injury. Pretreatment with PUE reversed the elevated VDAC1 protein levels, while Fer‐1 pretreatment did not result in a significant alteration of VDAC1 protein levels following I/R injury (Figure 2A,D).

In summary, the findings of our study indicate that pretreatment with PUE effectively suppresses ferroptosis in cardiomyocytes subjected to I/R injury, potentially through modulation of VDAC1 expression.

### 3.3 PUE Mitigates Erastin‐Induced Ferroptosis in H9c2 Cells

**FIGURE 3:**
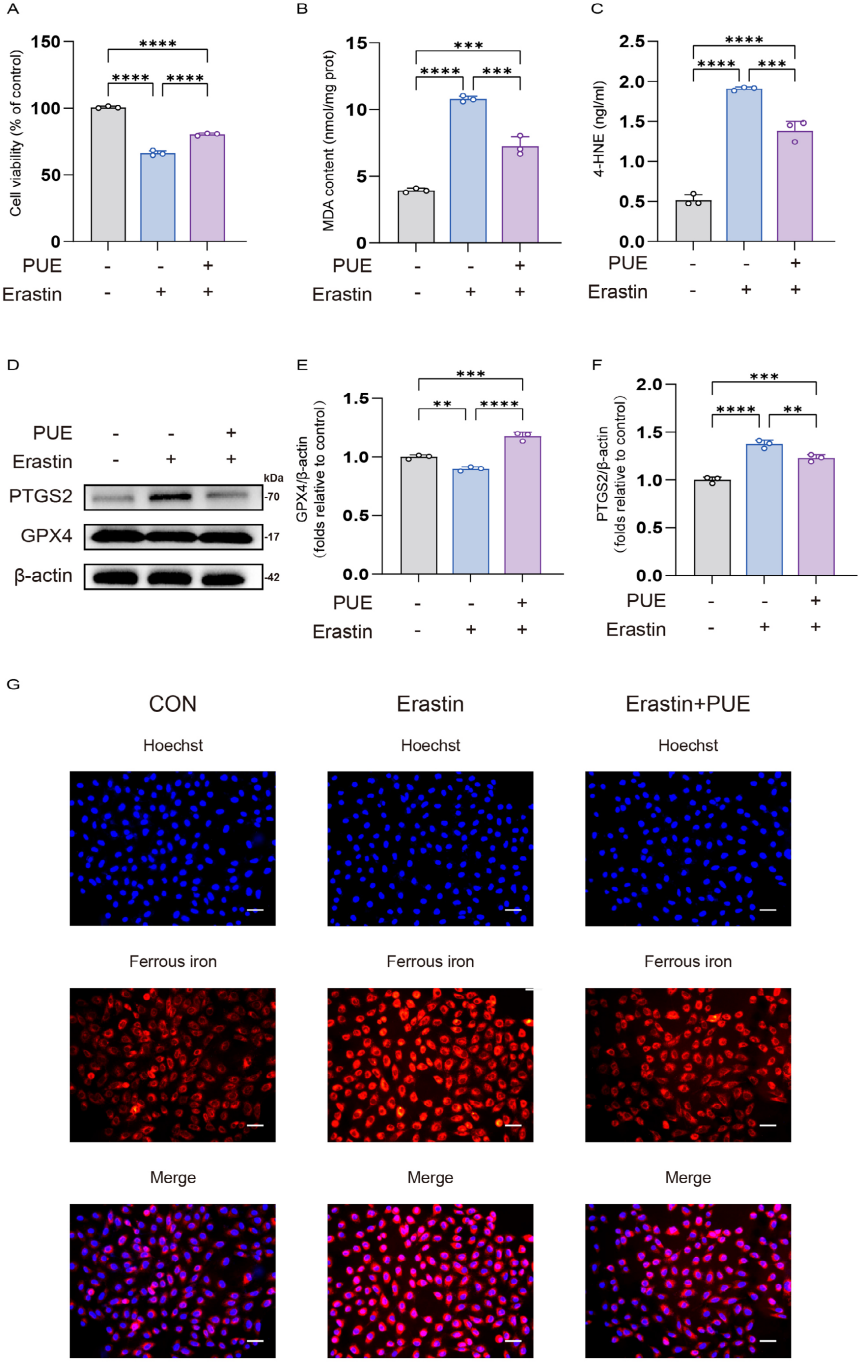
PUE mitigates erastin‐induced ferroptosis in H9c2 cells (A) Histogram of CCK‐8 detected the cell viability after treatment of each group separately. (B) MDA level after treatment of each group separately. (C) 4‐HNE level after treatment of each group separately. (D) Western blot detection of PTGS2 protein and GPX4 protein expression after treatment of each group separately. (E, F) Histogram of PTGS2 protein and GPX4 protein expression. (G) Intracellular Fe^2+^ was assessed by FerroOrange in H9c2 cells after treatment of each group separately. (magnification, ×200; scale bar, 400 μm). Data are expressed as the mean ± SD (*n* = 3). **p < 0.01, ***p < 0.001, ****p < 0.0001.

To elucidate the protective effects of PUE on myocardial against I/R injury through the inhibition of ferroptosis, we employed erastin, a well‐established inducer of ferroptosis. The results indicate that erastin pretreatment significantly diminishes the viability of H9c2 cells. However, this effect is reversed upon the administration of PUE pretreatment (Figure 3A). Given that ferroptosis is characterised by lipid peroxidation and iron overload, we assessed the levels of MDA, 4‐HNE and divalent iron in each experimental group. The results demonstrated that erastin treatment significantly increased the levels of MDA, 4‐HNE and divalent iron, while pretreatment with PUE effectively reversed these effects (Figure 3B,C,G; Figure S2A). Furthermore, we assessed the protein levels of the classical ferroptosis markers, PTGS2 and GPX4. Erastin treatment resulted in elevated PTGS2 levels and reduced GPX4 levels, both of which were also reversed by PUE pretreatment (Figure 3D–F).

### 3.4 PUE Inhibits Ferroptosis of H/R Induced H9c2 Cells by Mediating VDAC1

LDH activity and cell viability serve as crucial indicators for evaluating cellular damage [33]. In this study, H9c2 cells were pretreated for 48 h using different concentrations of PUE. Subsequently, cell survival was assessed through a CCK‐8 assay to elucidate the potential toxicity of PUE at varying concentrations on H9c2 cells. Our findings indicate that there was no significant toxic impact observed on H9c2 cells following treatment with different concentrations of PUE (Figure S1A). Subsequently, an H/R injury model was established in H9c2 cells, followed by pretreatment with varying concentrations of PUE in order to investigate the protective effects on cardiomyocytes. The results from the CCK‐8 assay indicated that PUE significantly improved cell viability in a concentration‐dependent manner post‐H/R injury. However, cell viability started to decrease once the concentration of PUE exceeded 10 μM (Figure S1B). Consequently, a concentration of 10 μM PUE was selected for further experimentation.

**FIGURE 4:**
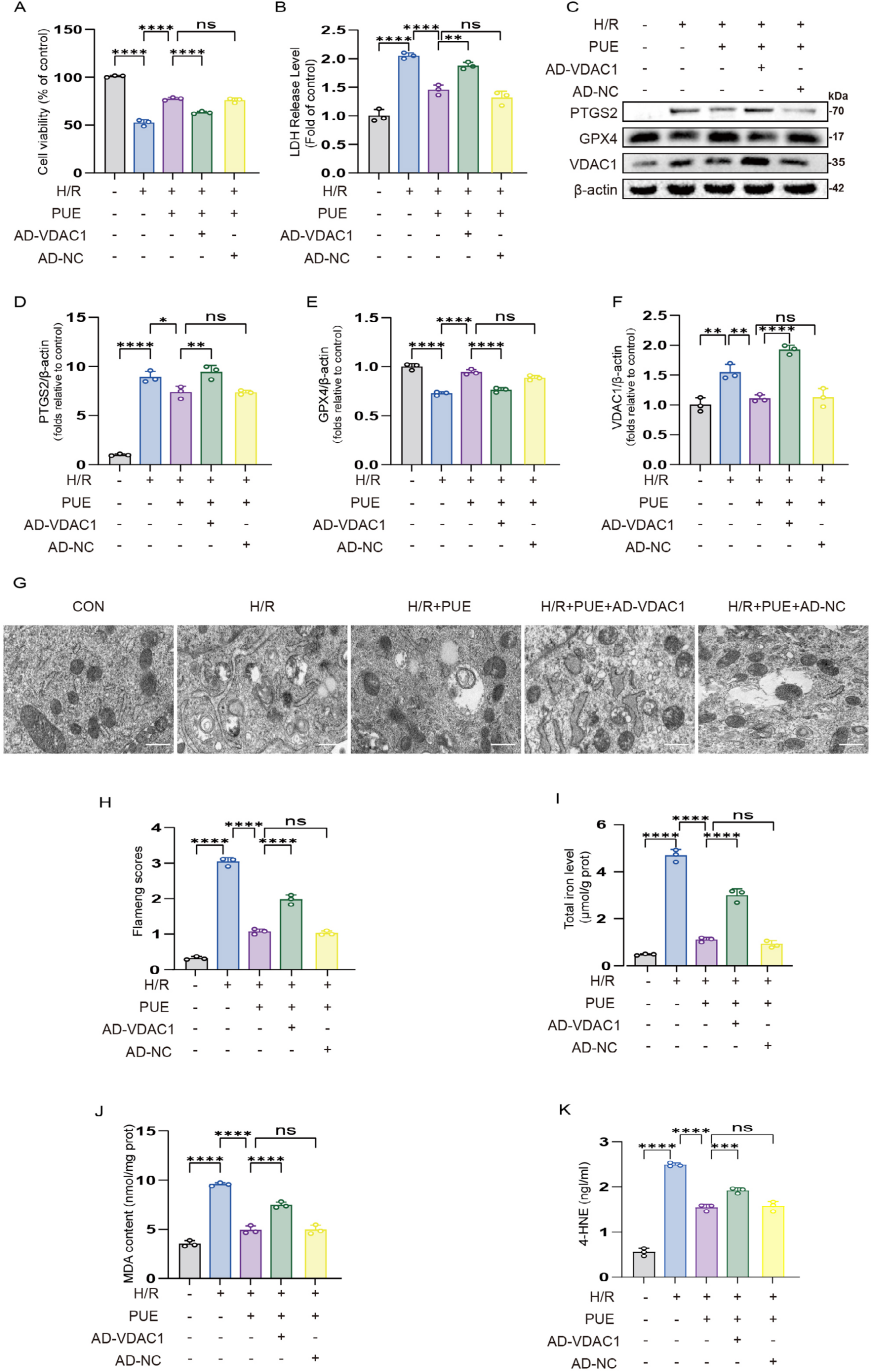
PUE inhibits ferroptosis of H/R‐induced H9c2 cells by mediating VDAC1. (A) Histogram of CCK‐8 detected the cell viability in H/R‐induced cells after treatment of each group separately. (B) Histogram of LDH activity in H/R‐induced cells after treatment of each group separately. (C) Western blot detection of PTGS2 protein, GPX4 protein and VDAC1 protein expression in H/R‐induced cells after treatment of each group separately. (D, E, F) Histogram of PTGS2 protein, GPX4 protein and VDAC1 protein expression. (G) Transmission electron microscopy images of H9c2 cells (magnification, ×6,000; scale bar, 1 μm). (H) Histogram of Flameng scores. (I) Total iron level in H/R‐induced cells after treatment of each group separately. (J) MDA level in H/R‐induced cells after treatment of each group separately. (K) 4‐HNE level after treatment of each group separately. Data are expressed as the mean ± SD (*n* = 3). ns, nonsignificant, *p < 0.05, **p < 0.01, ****p < 0.0001.

To further explore the inhibitory impact of PUE on ferroptosis in H/R injury through the VDAC1 pathway, we conducted adenoviral transfection to overexpress VDAC1 in H9c2 cells. Our findings indicate that PUE pretreatment enhanced cell survival following H/R injury and reduced LDH levels. Importantly, AD‐VDAC1 pretreatment effectively counteracted the protective effects of PUE (Figure 4A,B). Additionally, western blot analysis of PTGS2 and GPX4 revealed consistent results with in vivo experiments following PUE pretreatment, with AD‐VDAC1 pretreatment reversing these observed changes (Figure 4C–F).

**FIGURE 5:**
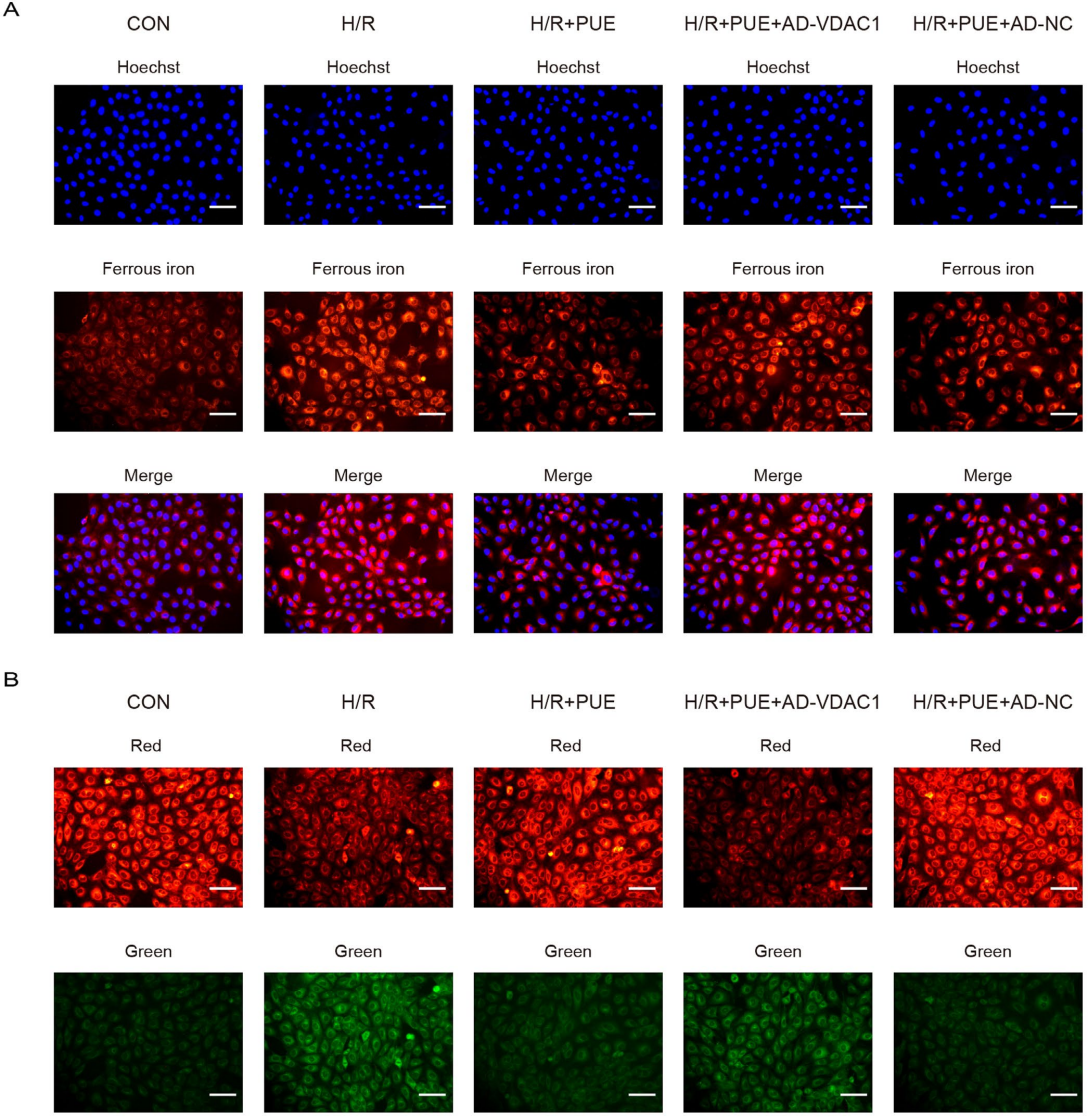
PUE inhibits ferrous iron generation and lipid oxidation in H/R‐induced H9c2 cells by mediating VDAC1. (A) Intracellular Fe^2+^ assessed by FerroOrange in H/R‐induced H9c2 cells after treatment of each group separately (magnification, ×200; scale bar, 400 μm). (B) Representative images of C11‐BODIPY (581/591) in H/R‐induced H9c2 cells after treatment of each group separately (magnification, ×200; scale bar, 400 μm). Data are expressed as the mean ± SD (*n* = 3).

Ferroptosis is primarily distinguished by lipid peroxidation and the build‐up of polyunsaturated fatty acids, which impacts mitochondrial morphology. In this research, mitochondrial morphology was examined through transmission electron microscopy, total iron and MDA levels were analysed in H9c2 cell lysates, and Fe^2+^ and lipid peroxidation levels were observed using fluorescence microscopy. The findings indicated distorted mitochondrial morphology, decreased mitochondrial cristae and notably elevated Flameng scores following H/R injury. Pretreatment with PUE mitigated the changes in mitochondrial morphology, while AD‐VDAC1 negated the effects of PUE pretreatment (Figure 4G,H). Meanwhile, PUE pretreatment showed the same results as the in vivo experiments in reducing total iron and MDA content, and it is noteworthy that AD‐VDAC1 treatment similarly reversed the above effects of PUE (Figure 4I,J). The measurement of 4‐HNE levels indicated that H/R significantly increased 4‐HNE concentrations. Pretreatment with PUE effectively reduced 4‐HNE levels, whereas the concurrent administration of AD‐VDAC1 reversed this reduction (Figure 4K). In addition, Fe^2+^ and lipid peroxidation levels were significantly elevated after H/R injury, an effect that was neutralised by PUE pretreatment, and the protective effects of PUE were reversed by pretreatment with AD‐VDAC1 (Figure 5A,B). A negative control group, AD‐NC, was included in each experiment to demonstrate that adenovirus did not affect cell functions.

To elucidate the role of VDAC1 in H/R, we conducted experiments involving the overexpression of VDAC1 under both H/R and non‐H/R conditions. The findings indicated that overexpression of VDAC1 in the absence of H/R did not result in statistically significant alterations in iron death‐related markers. However, when VDAC1 was overexpressed following H/R treatment, there was a notable increase in PTGS2 levels and a concomitant decrease in GPX4 levels, thereby exacerbating H/R‐induced ferroptosis (Figure S3A–D).

Overall, these findings indicate that PUE has the potential to attenuate ferroptosis in H9c2 cells following H/R injury through VDAC1.

### 3.5 PUE Pretreatment Improves Redox Homeostasis and Inhibits Apoptosis in H9c2 Cells After H/R Injury

**FIGURE 6:**
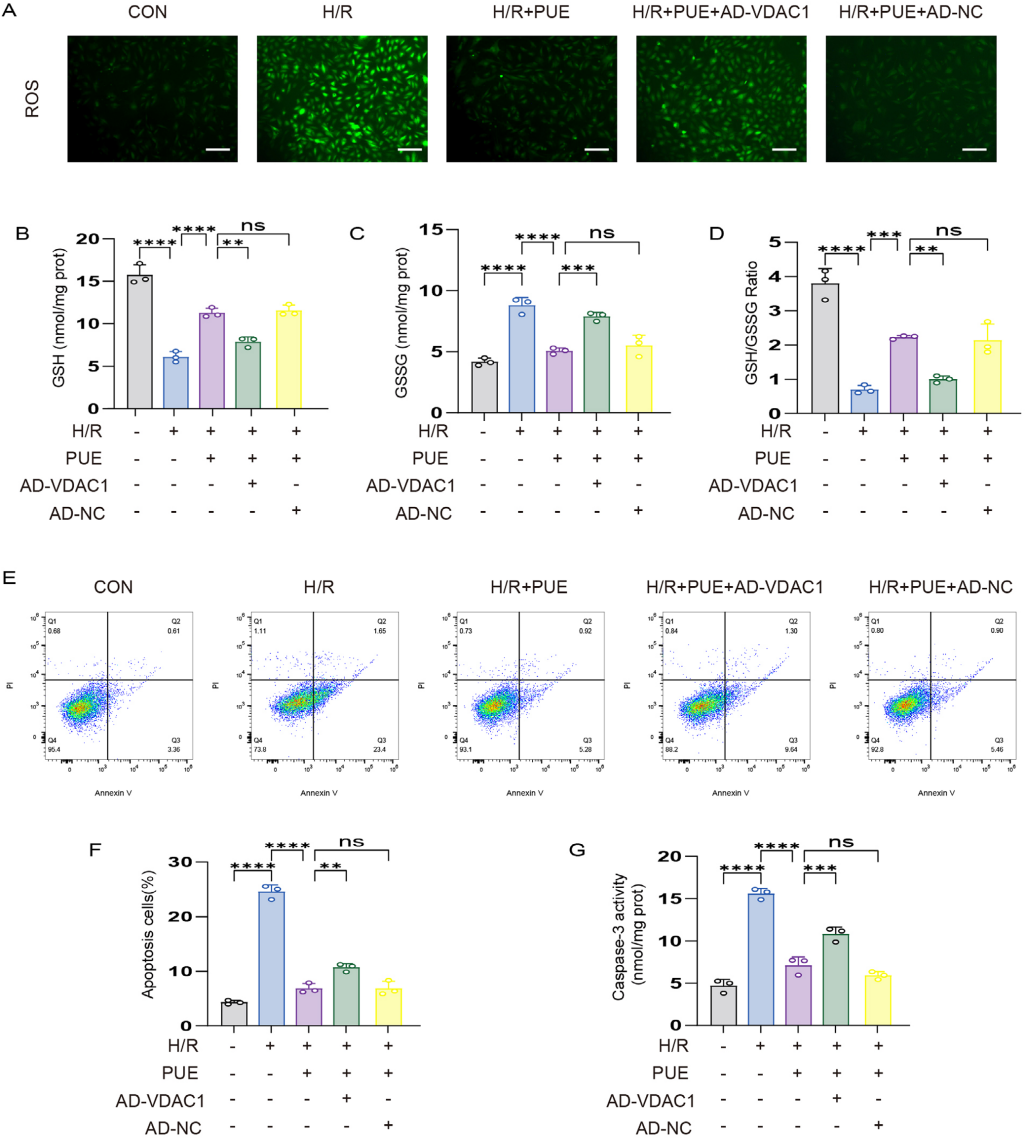
PUE pretreatment improves redox homeostasis and inhibits apoptosis in H9c2 cells after H/R injury. (A) DCFH‐DA stained images for detection of ROS (magnification, ×200; scale bar, 50 μm). (B) Histogram of GSH. (C) Histogram of GSSG. (D) Histogram of GSH and GSSG ratios. (E) Apoptotic rate measured by Annexin V‐FITC/PI detected by flow cytometry. (F) Histogram of apoptotic rate. (G) Histogram of caspase 3. Data are expressed as the mean ± SD (*n* = 3). ns, nonsignificant, **p < 0.01, ***p < 0.001, ****p < 0.0001.

Our study conducted a detailed analysis of the redox levels in each experimental group, demonstrating that pretreatment with PUE effectively suppressed ROS production in H9c2 cells subjected to H/R injury (Figure 6A). This injury resulted in a decrease in GSH activity, an increase in GSSG levels and a reduction in the GSH/GSSG ratio, all of which were mitigated by PUE pretreatment. Furthermore, the protective effects of PUE were partially negated by AD‐VDAC1 (Figure 6B–D). We also examined apoptosis‐related indexes and found that apoptosis rate and caspase‐3 activity were significantly increased after H/R injury, and PUE pretreatment reduced apoptosis rate and caspase‐3 activity in H9c2 cells, and the same AD‐VDAC1 pretreatment reversed the protective effect of PUE (Figure 6E–G).

In conclusion, PUE pretreatment inhibited apoptosis as well as restored redox homeostasis in H/R‐injured H9c2 cells, and the above protective effects were dependent on VDAC1 reduction.

### 3.6 PUE Pretreatment Attenuates Mitochondrial Dysfunction in H9c2 Cells After H/R Injury

**FIGURE 7:**
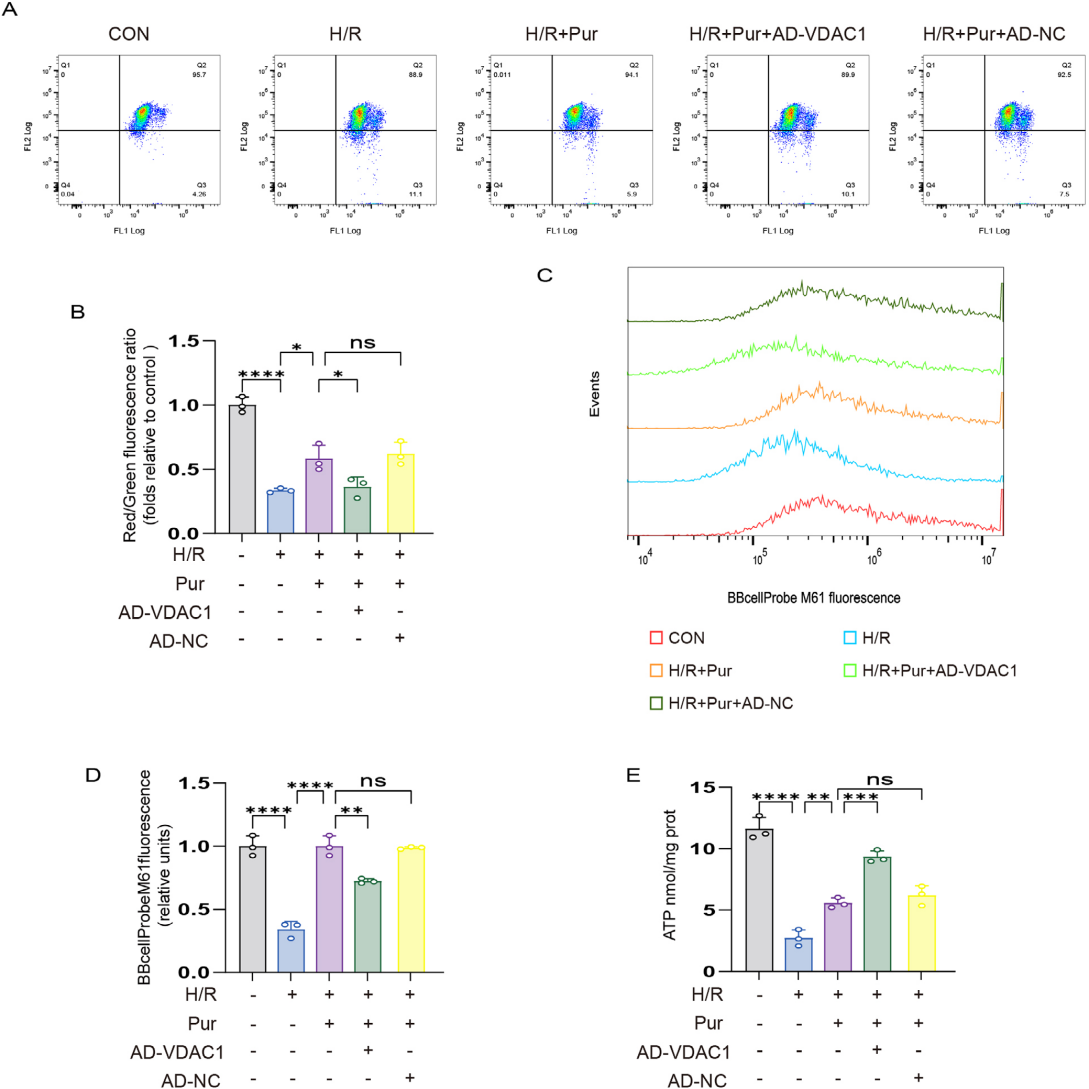
PUE pretreatment attenuates mitochondrial dysfunction in H9c2 cells after H/R injury. (A) MMP levels detected by JC‐1 in H9c2 cells by red/green fluorescence ratio. (B) Histogram of red/green fluorescence ratio. (C) Fluorescent probe BBcellProbe M61 indicating mPTP opening was detected by flow cytometry. (D) Histogram of mPTP flow cytometry results. (E) Histogram of ATP. Data are expressed as the mean ± SD (*n* = 3). ns, nonsignificant, *p < 0.05, **p < 0.01, ***p < 0.001, ****p < 0.0001.

Recent studies have shown that oxidative stress due to MI/RI is a major cause of impairment of mitochondrial function further aggravating MI/RI. We used JC‐1 and mPTP method probes to detect MMP and permeability transition, respectively. The results revealed that the red/green fluorescence ratio and green fluorescence were significantly reduced in the H/R group compared with the control group, whereas the opposite was observed in the H/R + PUE group. Interestingly, the addition of AD‐VDAC1 negated the protective effect of PUE pretreatment (Figure 7A–D).

A decrease in ATP levels, the main source of energy in cells, was noted after H/R injury. Nevertheless, pretreatment with PUE partially reinstated ATP levels, while AD‐VDAC1 reversed the aforementioned outcomes (Figure 7E).

**FIGURE 8:**
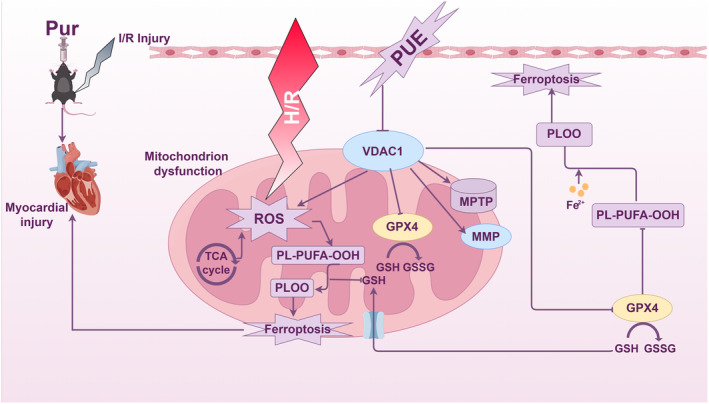
Schematic diagram of the mechanism by which PUE protects myocardium from I/R injury through VDAC1.

The results showed that PUE pretreatment attenuated mitochondrial dysfunction and restored mitochondrial energy metabolism, and these protective effects were dependent on VDAC1 (Figure 8).

## 4 Discussion

MI/RI is a significant contributor to mortality in cases of acute myocardial infarction, with ROS burst and mitochondrial dysfunction identified as primary mechanisms of myocardial damage. Research findings suggest that targeting oxidative stress and mitochondrial function may hold promise in mitigating ischaemia–reperfusion injury. In the present study, we showed that PUE preconditioning inhibited oxidative stress via VDAC1, improved energy metabolism and mitochondrial function during MI/RI and inhibited ferroptosis to protect cardiomyocytes from I/R injury. While numerous studies have shown the cardioprotective effects of PUE preconditioning against MI/RI, the precise underlying mechanism remains unclear. Our investigation into ferroptosis following MI/RI and its impact on mitochondrial function offers novel insights into the protective mechanisms against MI/RI.

Recent research has indicated that ferroptosis, a recently recognised form of programmed cell death, plays a significant role in the pathogenesis of MI/RI. It is characterised by heightened ROS generation, accumulation of Fe^2+^ and aberrant lipid metabolism [34]. Cai et al. [10] demonstrated that ferroptosis was the predominant mode of cell death in cardiomyocytes following 24 h of reperfusion, and further elucidated that Alox15 exacerbated ferroptosis, thereby aggravating MI/RI. In the current study, we aimed to mitigate MI/RI by inhibiting ferroptosis. Consequently, we employed a 24 h reperfusion protocol in our experimental design. Chen et al. [35] have shown that FOXC1 transcriptionally upregulates ELAVL1 and induces ferroptosis in MI/RI by regulating autophagy. Recent studies have shown that several traditional Chinese medicine compounds that target ferroptosis have emerged as a novel approach to protecting the myocardium from I/R injury. For instance, Yao et al. discovered that isoglycyrrhizin could mitigate MI/RI in mice by modulating the Nrf2/HO‐1/SLC7a11/GPX4 axis [36], while Yang et al. demonstrated that pretreatment with *Rhodiola rosea* glycoside could alleviate ferroptosis following ischaemia/reperfusion injury through AMPKα2 activation [37]. However, whether PUE inhibits MI/RI by targeting ferroptosis is not yet known.

PUE, a flavonoid compound, predominates in the root of *Pueraria lobata* [38]. Prior research has demonstrated that PUE possesses the ability to mitigate MI/RI through anti‐inflammatory, antioxidant and antiapoptotic mechanisms. More recent investigations have revealed that PUE can confer protective benefits in various diseases by inhibiting ferroptosis. In a study conducted by Song et al., it was observed that PUE could mitigate retinal iron accumulation induced by iron overload through the inhibition of Nrf2‐mediated ferroptosis [39]. Hou et al. discovered that PUE exhibits potential to mitigate excessive extracellular matrix accumulation by inhibiting ferroptosis in diabetic nephropathy [40]. The potential of PUE in inhibiting MI/RI through ferroptosis inhibition remains uncertain. Our hypothesis posits that PUE could mitigate MI/RI by inhibiting ferroptosis. Our findings demonstrate that PUE effectively reduced serum CK‐MB and LDH levels, enhanced cardiac function parameters and decreased levels of MDA and total iron in mice. Treatment with Fer‐1, a ferroptosis inhibitor, demonstrated similar protective effects. Through analysis of key indicators of ferroptosis, it was confirmed that PUE effectively mitigated I/R injury by suppressing ferroptosis.

VDAC1 is a protein located in the outer mitochondrial membrane that plays a crucial role in the tricarboxylic acid cycle by facilitating the transport of calcium ions and metabolites, such as ATP, across the outer mitochondrial membrane [41]. A study suggests that the HNF4A‐BAP31‐VDAC1 axis synchronises the regulation of gastric cancer cell proliferation and ferroptosis [42]. Our previous study also showed that resveratrol protects cardiomyocytes from I/R‐induced ferroptosis by inhibiting the VDAC1/GPX4 pathway [31]. In the present study, we confirmed that PUE inhibition of MI/RI‐induced ferroptosis was dependent on VDAC1. Cardiomyocytes exhibit a high concentration of mitochondria as a result of their elevated energy requirements. The mitochondrial membrane protein VDAC1 plays a significant role in mitochondrial function. Previous research has indicated that mitochondria could serve as potential therapeutic targets for cardiomyopathy [43]. Nevertheless, the specific mechanism by which PUE may enhance mitochondrial function following myocardial ischemia–reperfusion through VDAC1 remains unclear. Our study findings indicate that PUE can enhance mitochondrial function following MI/RI, with this protective effect being contingent upon the suppression of VDAC1. A limitation of the present study is that H9c2 cells, which are derived from rat embryonic hearts and retain numerous characteristics of cardiac myocytes, do not exhibit the functional maturity of fully developed cardiomyocytes.

In summary, existing research supports the notion that MI/RI leads to significant damage in cardiomyocytes via the activation of ferroptosis. It has been demonstrated that PUE can mitigate this phenomenon by suppressing ferroptosis through the downregulation of VDAC1, thereby preserving mitochondrial function integrity. These mechanisms collectively serve to safeguard the myocardium against the deleterious effects of MI/RI.

## Author Contributions

**Fajia Hu:** conceptualization (equal), data curation (equal), investigation (equal), methodology (equal), software (equal), writing – original draft (equal). **Tie Hu:** conceptualization (equal), data curation (equal), methodology (equal), writing – original draft (equal). **Andi He:** conceptualization (equal), data curation (equal), investigation (equal), methodology (equal), writing – original draft (equal). **Yong Yuan:** data curation (equal), software (equal). **Xiuqi Wang:** formal analysis (equal), methodology (equal), software (equal). **Chenchao Zou:** methodology (equal), software (equal). **Yamei Qiao:** data curation (equal), software (equal). **Huaihan Xu:** data curation (equal), software (equal). **Lanxiang Liu:** data curation (equal), methodology (equal). **Qun Wang:** conceptualization (equal), funding acquisition (equal), validation (equal), writing – review and editing (equal). **Jichun Liu:** conceptualization (equal), validation (equal), visualization (equal), writing – review and editing (equal). **Songqing Lai:** conceptualization (equal), funding acquisition (equal), investigation (equal), supervision (equal), validation (equal), writing – original draft (equal), writing – review and editing (equal). **Huang Huang:** conceptualization (equal), funding acquisition (equal), project administration (equal), supervision (equal), validation (equal), visualization (equal), writing – review and editing (equal).

## Conflicts of Interest

The authors declare no conflicts of interest.

## Supporting information


**FIGURE S1.** PUE protects H9c2 cells from injury caused by H/R.
**FIGURE S2.** Relative fluorescence intensities of ferrous iron in H9c2 cells.
**FIGURE S3.** Overexpression of VDAC1 is highly correlated with H/R‐induced H9c2 cell injury.

## Data Availability

The raw data supporting the conclusions of this article will be made available by the authors, without undue reservation, to any qualified researcher.
