# Supplementary material for: Puerarin Protects Myocardium From Ischaemia/Reperfusion Injury by Inhibiting Ferroptosis Through Downregulation of VDAC1
Source: J Cell Mol Med. 2024 Dec 27;28(24):e70313. doi: 10.1111/jcmm.70313 (PMC11680193; doi:10.1111/jcmm.70313)
Supplement: Supplementary file 1 — FIGURE S1. PUE protects H9c2 cells from injury caused by H/R. FIGURE S2. Relative fluorescence intensities of ferrous iron in H9c2 cells. FIGURE S3. Overexpression of VDAC1 is highly correlated with H/R‐induced H9c2 cell injury. [file JCMM-28-e70313-s001.docx]

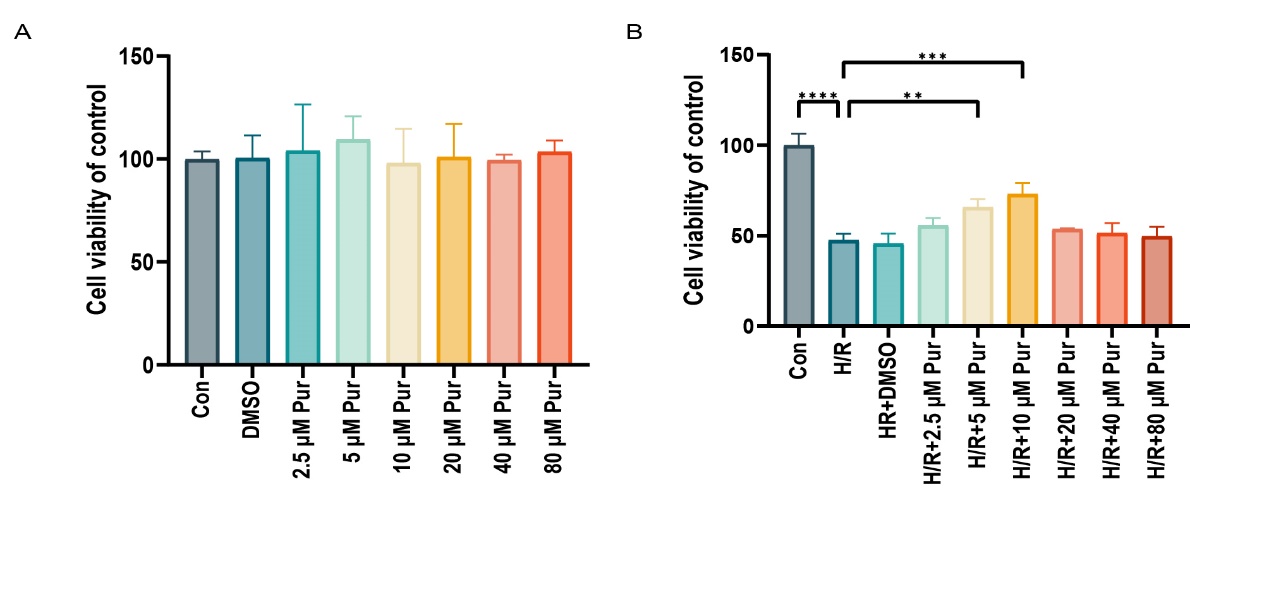


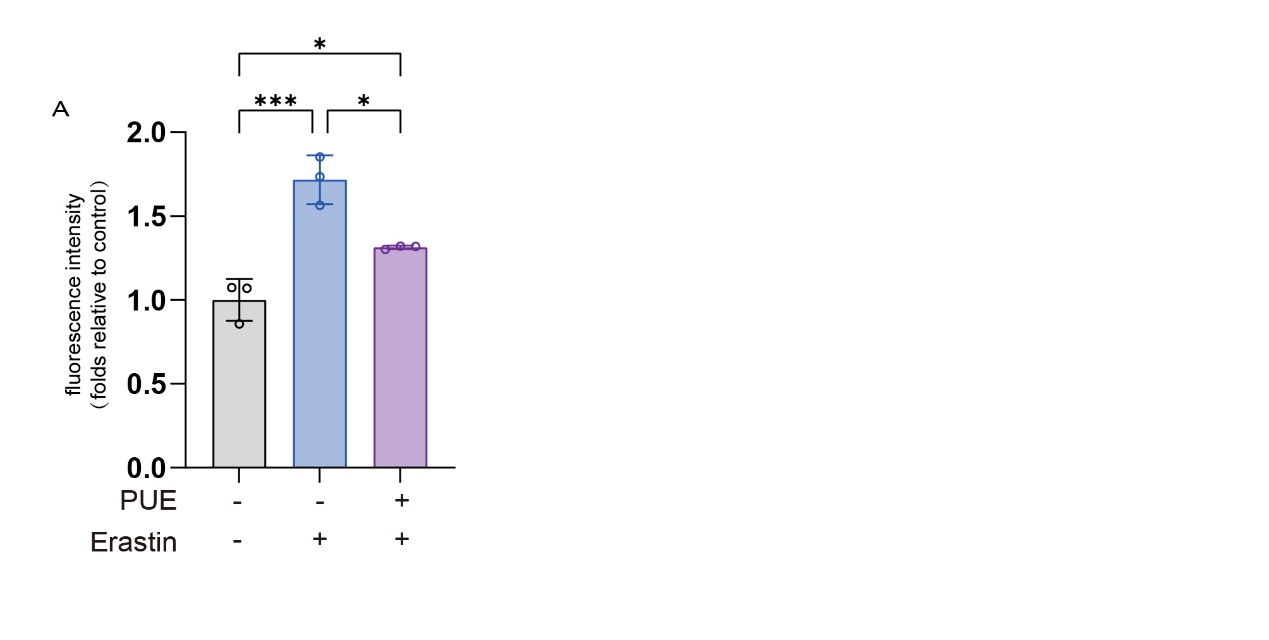
FIGURE S1: PUE protects to H9c2 cells from injury caused by H/R. (A) Histogram of CCK‑8 detected the cell viability when the different concentrations of PUE were induced. (B) Histogram of CCK‑8 detected the cell viability in H/R‑induced cells after different concentrations of PUE. Data are expressed as the mean ± SD (n = 3). **P<0.01, ***P<0.001, ****P<0.0001.

FIGURE S2: Relative fluorescence intensities of ferrous iron in H9c2 cells. (A) Histogram of fluorescence intensities. Data are expressed as the mean ± SD (n =3). *P < 0.05, ***P<0.001.


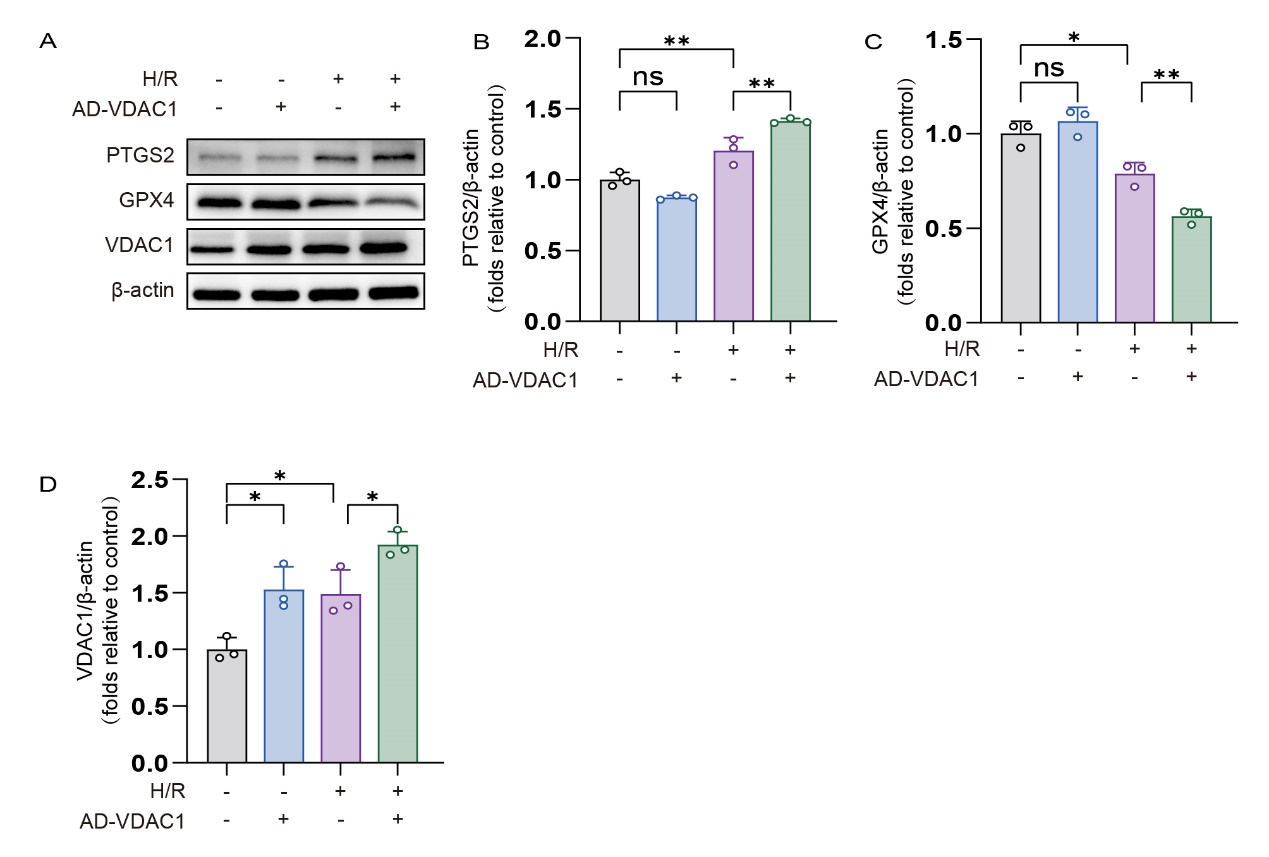
FIGURE S3: Overexpression of VDAC1 is highly correlated with H/R-induced H9c2 cell injury. (A)Western blot detection of PTGS2 protein, GPX4 protein and VDAC1 protein expression after treatment of each group separately. (B, C) Histogram of PTGS2 protein, GPX4 protein and VDAC1 protein expression. Data are expressed as the mean ± SD (n =3). ns, nonsignificant, *P < 0.05, **P<0.01.
